# Editorial: Innovative biologics in the battle against bacterial pathogens: disrupting pathogenesis with precision

**DOI:** 10.3389/fmicb.2026.1957108

**Published:** 2026-08-18

**Authors:** Valeria Szijarto, Gabor Nagy, Adriana Badarau, Christine Tkaczyk

**Affiliations:** 1 Independent Researcher, Sopron, Hungary; 2 CEBINA GmbH, Vienna, Austria; 3 BioNTech R&D, Vienna, Austria; 4 Early Infectious Disease, AstraZeneca, Gaithersburg, MD, United States

**Keywords:** AMR (antimicrobial resistance), bacterial infection, bacteriophage, biologics, vaccine, monoclonal antibody

Antibiotic resistance is a growing global crisis that represents a systemic threat to public health and a substantial economic burden on healthcare systems, emphasizing the need for innovative strategies to combat infectious diseases, particularly those caused by multi-drug resistant (MDR) bacteria. At the same time, numerous Gram-positive and Gram-negative pathogens remain to pose a substantial burden on healthcare systems by driving outbreaks and complicating clinical management regardless of their antibiotic resistance profiles. Recent technological advances have opened new avenues for a target-specific intervention through biologics—including vaccines, bacteriophages, lysins, monoclonal antibodies and *in vivo* expressed biologics—with the potential to disrupt bacterial pathogenesis at the molecular level and offer a more sustainable defense than traditional, broad spectrum antibiotics.

The eight articles in this Research Topic come from contributors across the globe, including countries like China, USA, Poland, and Italy emphasizing the need for global responses against global threats. They highlight innovative research on combatting bacterial infections through the development of a variety of next-generation biologics including vaccines, phages, mAb-based approach and other biologics.

Two articles describe novel vaccine candidates against drug-resistant bacteria. Zhou et al. further improved a previously described live attenuated vaccine to target non-typhoidal *Salmonella* infections by creating a strain that is deficient in lipopolysaccharides (LPS) and lacks specific immune evasion genes (*steD* and *yejAE*). With their approach the scientists simultaneously increased immunogenicity of conserved outer membrane proteins and restored the MHC-I dependent antigen presentation. The study highlights how this specific combination of deletions allows the vaccine to trigger cytotoxic T-cell response and provides significant cross-protection against multiple serotypes as well. Although antibiotic resistance is of limited concern in *Streptococcus mutans*, its role in dental caries and the associated high medical burden would make an effective vaccine largely beneficial. Zhou et al. provide proof-of-concept for an mRNA-LNP vaccine against *S. mutans*. Recognizing that traditional protein vaccines often produce transient mucosal immune response, the authors engineered an mRNA sequence that encodes the protein antigen c and fused with a human IgG Fc domain to exploit natural transport receptors in the mucosal lining. Combining a systemic intramuscular priming with a local intranasal boost the vaccine triggered potent and lasting secretory IgA (sIgA) antibody response in saliva that reduced biofilm formation *in vitro* and reduced moderate tooth decay in a rat model. Ultimately, the paper suggests that this Fc-fusion strategy can be leveraged to enhance mucosal immunity against a wide variety of oral and respiratory pathogens.

Three papers in the topic explore the potential of bacteriophages as antibacterial agents. Cheng et al. reviewed literature about phages targeting *Acinetobacter baumannii* by outlining how phages employ diverse mechanisms to overcome bacterial defenses that standard antibiotics cannot overcome. Beyond direct killing, the authors highlight how exposure to bacteriophages can force an evolutionary trade-off, where bacteria become less virulent or regain sensitivity to antibiotics as they attempt to develop phage resistance. The paper concludes by discussing the future of the field, advocating for innovative bacteriophage engineering, the use of artificial intelligence in design, and more flexible regulatory frameworks to facilitate personalized clinical applications. In the paper of Necel et al. the activity of the novel bacteriophage vB_Pa_AN-12 was explored against multi-drug resistant *Pseudomonas aeruginosa* clinical isolates. The scientists confirmed the high specificity of the phage to *P. aeruginosa* with a considerable coverage and showed that it can elicit rapid killing, while having no negative impact on three different human cell lines. Once evaluated in animal models, this phage may demonstrate therapeutic potential, especially for the treatment of skin infections. The third paper, from Feng et al. identified novel phages against methicilling resistant *Staphylococcus pseudintermedius* (MRSP), the predominant bacterium causing canine pyoderma. Besides the *in vitro* antibacterial activity, one phage even showed significant benefit in a rabbit pyoderma model, especially when combined with othewise ineffective antibiotic. Although human infections caused by MRSP following interspecies trasnmission are still rare, its multi-drug resistance represents a potential public health concern and emphasizes the importance of a one health approach to antibiotic resistance in general.

The novel anti-*Neisseria gonorrhoeae* mAb described by Mokhtary et al. advances our understanding of the cross-protection provided by the 4CMenB meningococcal vaccine against gonococcal infections. The authors isolated mAb_4 from vaccinated individuals and identified its molecular target as an outer membrane protein involved in membrane stability. Although mAb_4 exhibited no complement or phagocyte mediated bacterial killing, it holds promise as a diagnostic tool or as part of a mAb cocktail.

The Research Topic attracted two reviews about other biologics as alternatives against antibiotic resistant bacterial infections. Kosznik-Kwasnicka et al. provided an overview on the versatile role of lactoferrin in *Staphylococcus aureus* skin infections. The authors discussed how lactoferrin and its derived-peptides exert direct antibacterial effect, promote wound healing and how incorporation of lactoferrin into novel technological solutions, like hydrogels could confer protection against methicillin resistant *S. aureus* skin infection. Melo and Re reviewed potential host directed biologics against *Burkholderia pseudomallei*, the bacterium causing melioidosis. The authors provide a systematic overview of the harmful role of dysregulated inflammation and coagulation in melioidosis, with particular emphasis on the NLRP3 inflammasome, neutrophils and IL-1β. The authors have proposed that approved or investigational drugs targeting these host factors may represent promising therapuetic strategies against melioidosis.

Finally, we would like to extend our sincere appreciation to all the authors who contributed their original research to this Research Topic, as well as to the reviewers of all 13 submitted manuscripts for their insightful and constructive feedback. We are also grateful to the editorial team at *Frontiers in Microbiology* for their continuous support and for providing us with the opportunity to successfully host this Research Topic.

